# Transcutaneous Electrical Nerve Stimulation for Prevention and Treatment of Post-Herpetic Neuralgia: A Narrative Review

**DOI:** 10.7759/cureus.74416

**Published:** 2024-11-25

**Authors:** Alan D Kaye, Rahib K Islam, Victoria T Tong, Brynne E Tynes, Kelly R Sala, Brennan Abbott, Chandni R Patel, Isabella B Lentz, Raju Behara, Shilpadevi Patil, Uzayr Wasif, Sahar Shekoohi, Giustino Varrassi

**Affiliations:** 1 Department of Anesthesiology, Louisiana State University Health Sciences Center, Shreveport, USA; 2 School of Medicine, Louisiana State University School of Medicine, New Orleans, USA; 3 School of Medicine, Louisiana State University (LSU) Health Sciences Center New Orleans School of Medicine, New Orleans, USA; 4 School of Medicine, Louisiana State University Health Sciences Center, Shreveport, USA; 5 School of Medicine, Louisiana Health Sciences Center New Orleans School of Medicine, New Orleans, USA; 6 School of Medicine, St. George’s University, True Blue, GRD; 7 Department of Biology, The State University of New Jersey, New Brunswick, USA; 8 Department of Pain Medicine, Fondazione Paolo Procacci, Rome, ITA

**Keywords:** electrical nerve stimulation, neuropathic pain, post herpetic neuralgia, shingles, transcutaneous

## Abstract

The present investigation evaluated transcutaneous electrical nerve stimulation (TENS) for the non-pharmacological treatment of post-herpetic neuralgia (PHN). PHN is the most common complication of the Herpes Zoster virus, affecting one in every five patients with shingles, and presents as intense neuropathic pain that can persist for 90 days or longer after the initial onset of symptoms. Current pharmacological treatment options are mainly limited to symptom management, including oral medications such as tricyclic antidepressants and gabapentinoid preparations, as well as topical options such as lidocaine or capsaicin. TENS is a minimally invasive, non-pharmacological electrical nerve stimulation device currently approved for the treatment of neuropathic pain in adults, providing patients with an alternative or adjunct treatment option to medication with a reduced potential for unwanted side effects, drug-drug interactions or potentially life-threatening toxicity. Current indications for the use of TENS in the treatment of PHN are recommended only after therapeutic goals are attempted and unsuccessfully met by current mainstay medications. More research into the efficacy and safety of TENS for treating PHN should be conducted to clarify further its role as a mainstay treatment for this condition.

## Introduction and background

For most individuals living in the United States, “chickenpox” is well known as a classic, contagious childhood illness involving flu-like symptoms with the additional presentation of an itchy, herpetic rash. Chickenpox, caused by the varicella-zoster virus (VZV), is a self-limiting disease that presents with fever, headache, nausea, malaise, and a generalized herpetic rash that evolves into itchy, fluid-filled blisters over the disease course [1]. It generally dissipates within 4-7 days and can be treated with antivirals such as acyclovir. This used to be a prevalent childhood illness, with an average of 4 million new cases per year in the United States reported throughout the early 1990s [2]. At that time, hospitalizations averaged between 10,500 and 13,500 per year, with approximately 100-150 deaths, half of which being school-aged children [3]. With the development, implementation, and inclusion of the VZV vaccine into the United States’ routine childhood immunization schedule in 1995, annual chickenpox cases have since declined by more than 97%, with an estimated 91 million US cases of chickenpox prevented. However, for everyone who had been infected with VZV before the introduction of the vaccine and for those 150,000 US citizens who become newly infected with chickenpox each year, there remain potential long-term complications of the disease, namely shingles [4]. Shingles, or herpes zoster, is an illness caused by the reactivation of dormant VZV. As a result of the frequency of VZV infection before the vaccine development and popularization, there are around 1 million cases of Shingles in the United States each year, with an individual lifetime risk of 30% [5]. Shingles classically present as a unilateral, dermatomal rash with clear vesicles. As with VZV, herpes zoster can also be treated with antivirals such as acyclovir or valacyclovir. Herpes zoster comes with its unique complications; however, the most common is postherpetic neuralgia (PHN), which is a type of neuropathic pain following acute herpes zoster infection in the affected dermatome. This results from nerve damage secondary to herpes zoster reactivation, replication, and travel down the axons of the affected dermatome [6]. This complication arises in approximately one in five Shingles patients [5]. Patients describe the pain as an intense burning sensation that persists on average 90 days longer after initial herpes zoster reactivation. Current treatment options are limited and focus predominantly on symptom control [5]. Current treatments are primarily symptom-focused and include oral tricyclic antidepressants (TCA), such as amitriptyline and gabapentin, as well as topicals, such as lidocaine or capsaicin. The purpose of this narrative review, therefore, is to examine the potential role of TENS as an alternative or adjunctive therapy for PHN, a modality currently used in the treatment of chronic idiopathic neuropathic pain in adults [7].

## Review

Overview of postherpetic neuralgia

PHN is a common chronic neuropathic pain complication caused by VZV reactivation, also known as herpes zoster (HZ) or shingles. Neuralgia refers to pain resulting from nerve inflammation or damage. Herpes zoster presents with a painful vesicular rash in a band-like dermatomal distribution. The painful rash heals within a few months, but it is considered postherpetic neuralgia if it persists for more than three months following the acute herpes zoster episode [8]. PHN is characterized by intense, lancinating pain in a unilateral dermatomal pattern where a herpes zoster rash outbreak previously occurred [9]. The pain can be constant or intermittent and is variable among patients, often being described as burning, stabbing, shooting, aching, or throbbing [10].

Pathophysiology

VZV is a herpes virus that is the causative agent for varicella (chickenpox) and herpes zoster (shingles). The primary infection of VZV occurs in non-immune hosts and manifests as an asynchronous vesicular rash and skin lesions implicative of childhood exanthem varicella [11]. During this primary infection, the virus replicates and infects the sensory neurons through retrograde axonal transport from the skin [12]. Following infection, VZV enters its dormant phase, where it establishes latency in the neurons of the dorsal root ganglia [12]. In some individuals, VZV may reactivate and travel anterogradely through the axon to the innervated dermatome, resulting in the dermatomal rash pattern characteristic of HZ [12,13]. Although the underlying mechanism remains unclear, the reactivation of VZV is thought to be associated with a decline in cell-mediated immunity, often related to advancing age and physical or psychological stressors [14,15].

PHN results from peripheral nerve injury and signal changes in the central nervous system induced by the VZV virus [16]. VZV reactivation causes inflammation of the nerves under the skin at or near the area affected by the herpes zoster rash during migration. Damage to peripheral and central nerves induces the release of inflammatory mediators (bradykinin, prostaglandins, and proinflammatory cytokines and chemokines) from nociceptors located in spinal cord terminals, resulting in hyperexcitability and peripheral nerve sensitization [17]. Hyperactivity at peripheral nerve terminals results in a reduced threshold for action potentials and, therefore, heightened responses to pain [18]. Sustained sensory input from noxious stimuli induces synaptic plasticity and increased expression of pain receptors in the central nervous system, leading to central sensitization [17,19]. PHN impairs all sensory fiber groups, resulting in constant pain due to loss of nociceptive afferents and paroxysmal pain due to demyelination of large diameter Aβ fibers [20]. Central sensitization is responsible for pain perception independent of intensity, duration, or presence of noxious inputs in patients suffering from PHN [21].

Incidence and Prevalence

PHN is the most common and severe complication of herpes zoster [22]. The Centers for Disease Control and Prevention (CDC) estimates that the United States has approximately one million cases of herpes zoster annually, with an individual lifetime risk of 30% [23,24]. It is estimated that 5-20% of patients with herpes zoster will go on to develop PHN, with that number sharply rising to 60-75% after the age of 50 [9,25]. Although two vaccines for herpes zoster are available, the incidence of herpes zoster and PHN has been rising in recent decades in age groups for which the herpes zoster vaccines are not currently recommended [26]. From 1994 to 2018, there has been an annual enhancement of 3.1% (95% CI, 2.5-3.6%) in the incidence of herpes zoster in the United States, with the overall incidence rate of PHN being 57.5 cases per 100,000 person-years (95% CI, 56.0-59.0) [26]. The incidence of herpes zoster and PHN is uncommon in pediatric populations, especially in children vaccinated against VZV [27].

Risk Factors

A meta-analysis of 4,192 patients with HZ found that age [OR=1.59; 95% CI: (1.23, 2.04); Z=3.62; P<.001], acute severe pain in the herpes stage [OR=1.49; 95% CI: (1.08, 2.08); Z=2.39; P=.02], prodromal symptoms [OR=2.00; 95% CI: (1.16, 3.44); Z=2.48; P=.01], and severity [OR=2.40; 95% CI: (1.83, 3.14); Z=6.38; P<.001] were independent risk factors for PHN [16]. PHN has also been associated with hypertension, diabetes, coronary heart disease, and connective tissue diseases such as rheumatoid arthritis, Sjogren’s syndrome, and systemic lupus erythematosus [28]. The use of immunosuppressive treatments is a risk factor for PHN. Screening for independent risk factors of PHN and early clinical intervention may significantly reduce PHN's incidence rate and severity [16].

Transcutaneous electrical nerve stimulation (TENS)

The TENS unit consists of a battery-operated controlling circuit connected by wires to adhesive electrode pads placed on the skin. A TENS unit delivers electrical currents to areas of intact skin to stimulate the underlying nerves and provide non-invasive, non-pharmacological pain relief for various acute and chronic pain conditions [29]. TENS mediates its analgesic effects through both central and peripheral nervous system mechanisms. Different TENS techniques have alterations in intensity and frequency for users to selectively activate a population of nerve fibers and elicit individualized pain relief [29]. Standard methods of TENS delivery include conventional, acupuncture-like, burst mode, intense, and modulated TENS. Patients can safely self-administer TENS with no potential for toxicity, making it an excellent alternative for pharmacological pain relief [29]. If a TENS unit is placed in the wrong position, patients will not experience any pain relief and could potentially experience discomfort due to improper nerve stimulation [30]. In extreme cases, placing the electrodes incorrectly could lead to skin irritation or burns if the current is too concentrated on a small area of skin [30]. In the event of adverse effects, immediately remove the electrodes and reposition them or consult a healthcare professional for guidance on proper placement.

Affordability and Availability

Once a TENS device is purchased, it can be used continuously without ongoing costs, unlike pharmacological treatments. TENS therapy can also reduce costs associated with medication side effects and interactions. Long-term TENS therapy may be associated with significant reductions in pain medication utilization. Another advantage is the availability of TENS equipment. TENS devices are available over the counter in most countries. The ease of access reduces the need for frequent medical visits and provides a convenient option for managing pain.

Proposed mechanisms of TENS

Central Mechanisms of TENS

TENS stimulation of Aδ fibers leads to the activation of descending inhibitory pathways in the periaqueductal gray (PAG), rostral ventromedial medulla (RVM), and spinal cord, reducing hyperalgesia [30]. When activated, descending inhibitory pathways in the central nervous system release neurotransmitters such as endorphins, serotonin, acetylcholine, norepinephrine, and gamma-aminobutyric acid (GABA) that activate their respective receptors to mediate central effects [31]. Low- and high-frequency TENS stimulates μ- and δ-opioid receptors, respectively, to decrease nociceptive dorsal horn neuronal activity and ultimately lead to reductions in central excitability and sensitization [30]. Low-frequency TENS stimulates the release of serotonin, which acts on 5-HT2A and 5-HT3 serotonin receptors in the spinal cord to produce analgesic effects [32]. Low- and high-frequency TENS stimulates the release of acetylcholine, which acts on M1 and M3 receptors in the spinal cord to produce analgesic effects [33]. Low- and high-frequency TENS reduces algesia through its effects on GABAA receptors, and high-frequency TENS can be found.

Peripheral Mechanisms of TENS

TENS can produce analgesia by inhibiting nociceptor neurotransmission in the periphery [32]. TENS activates Aβ (large-diameter, low-threshold, non-noxious) fibers and Aδ (small-diameter, high-threshold, noxious) afferent fibers that send sensory information from the periphery to the central nervous system [34]. The antidromic activation of afferent fibers by TENS blocks the transmission of nociceptive information in peripheral nerves, preventing pain signals from reaching the spinal cord and brain [30]. The analgesia produced by TENS was not made in ɑ2-a adrenergic knockout mice, indicating that peripheral ɑ-2a receptors may play an essential role in the peripheral mechanism of TENS-mediated analgesia [35].

Classification of TENS units

Conventional TENS

Conventional TENS utilizes low-intensity, high frequency (50-100 Hz) electrical currents in short pulses (50-200 μs) to selectively activate large diameter, non-noxious Aβ fibers [34,36]. This form of TENS is often used to elicit segmental analgesia at dermatomal pain sites [37]. Patients are reported to experience rapid onset and offset of strong, non-painful paresthesia with minimal muscle activity [29]. Conventional TENS is the most commonly used technique, especially for patients suffering from acute/chronic superficial pain, such as pain caused by dermatological etiologies and labor [38]. Conventional TENS can be administered as needed for pain relief [38, 39].

Acupuncture-like TENS

Acupuncture-like TENS (AL-TENS) utilizes high-intensity, low-frequency (2-4 Hz) electrical currents in extended pulses (100-200 μs) to activate small diameter Aδ fibers [34,36] selectively. This form of TENS is often used to elicit extra segmental analgesia to myotomal pain sites, such as motor nerves and acupuncture trigger points [37]. Patients are reported to experience a possible delay in onset and offset of strong but comfortable paresthesia accompanied by muscle twitches [29]. AL-TENS is recommended for reducing longstanding deep pain, such as postoperative pain or chronic back pain [38,40]. Patients can administer AL-TENS 15-30 minutes at a time, up to three times daily, as needed for pain relief [40].

Intense TENS

Intense TENS utilizes high-intensity, high-frequency (100-150 Hz) electrical currents in long pulses (150-250 μs) to selectively activate noxious Aδ fibers [34,41]. This form of TENS is often used to elicit extra-segmental analgesia and peripheral nerve blockade [29]. Patients are reported to experience rapid onset and offset of painful paresthesia [29]. Intense TENS may help alleviate severe acute/chronic pain, such as pain related to cancer [38]. Intense TENS is also a counter-irritant in minor procedures such as wound dressing and suture removal [39]. Intense TENS should not be administered for more than five minutes at a time [39].

Burst Mode TENS

Burst Mode TENS combines low-frequency currents and high-frequency pulses to activate peripheral nerve fibers [31,42]. High-frequency electrical currents (approximately 100 Hz) are interrupted by low-frequency (1-4 Hz) bursts to stimulate both large diameter Aβ and small diameter Aδ fibers [42,43]. Burst mode TENS stimulates nerves in a more analogous pattern to physiologic sympathetic nerve activity, producing a more comfortable muscle contraction [42]. The muscle contraction elicited from burst mode TENS has produced a transient increase in local blood flow, which may help alleviate vascular resistance pain [42]. The administration of burst mode TENS is tailored to meet different patient needs.

Modulated TENS

Modulated TENS provides individualized pain relief for specific conditions by altering the temporal pattern of electrical pulses. Depending on the affliction it intends to treat, modulated TENS may have variations in pulse duration, frequency, and intensity. Modulated TENS has been clinically alleviating soft tissue pain [44]. Several clinical trials have been conducted on TENS therapy for treating PHN. In one randomized control clinical trial, 20 subjects with a previous diagnosis of PHN and a history of resistance to other treatments were randomly assigned either TENS or a sham device [45]. After three treatment sessions, those assigned the sham device were switched to a TENS device for the last three treatments. Pain was self-reported by subjects using a standard neuropathic pain scale score. Six subjects from the TENS group and eight from the sham/TENS group reported at least a 15% decrease in pain with a range of 18-92% (p<.05). Furthermore, the reduction of symptoms averaged 60.6% in these 14 subjects (p<.001). These results show a significant decrease in pain in most subjects who received at least three TENS treatment sessions.

Another randomized control clinical trial investigated the use of pregabalin along with TENS therapy for PHN [46]. A total of 30 patients aged 50-80 with a history of neuropathic pain related to PHN were randomly sorted into the pregabalin/TENS group or pregabalin/TENS placebo group. Progress was measured at eight outpatient visits using a Visual Analog Scale (VAS), Short Form-McGill Pain Questionnaire, and sleep interference questionnaire. Both groups showed decreases in all parameters by the end of the study. However, the pregabalin/TENS group showed a significantly more significant reduction in VAS than the pregabalin/TENS placebo group. Therefore, the authors concluded that better outcomes are achieved with the addition of TENS therapy to pregabalin treatment for PHN.

One randomized control clinical trial compared TENS therapy with cobalamin or lidocaine injections for the treatment of PHN [47]. A total of 90 subjects at least 50 years old with PHN and a pain score of 4 or higher were randomly assigned TENS treatment as well as either cobalamin, lidocaine, or both. Subjects receiving TENS and cobalamin injections demonstrated significant improvement after eight weeks, showing the efficacy of the combination therapy in treating PHN. Another double-blinded randomized control clinical trial compared TENS to pulsed electromagnetic field therapy (PEMFT) [48]. Fifty-six subjects with PHN of the sciatic nerve were randomly divided into the TENS or PEMFT group. Both groups completed conventional physical therapy and their respective interventions in 20-minute sessions three times weekly for eight weeks. Progress was measured using the VAS and carbamazepine intake (CMI) before and after treatment. Both measurements decreased significantly in all groups (p<.001), with no significant differences (p>.05). This suggests that TENS, PEMFT treatment, and physical therapy are effective and comparable in managing PHN (p<.05). Overall, TENS therapy seems to be safe and effective in treating PHN-related neuropathic pain and is similar to other interventions. It may be combined with other agents for a multimodal approach to achieve optimal outcomes (Table 1).

**Table 1:** Table showcasing TENS duration and frequency of use. TENS: Transcutaneous electrical nerve stimulation Sources: [31, 34-36, 44]

TENS	Parameters	Physiological Intention	Uses
Conventional TENS	Low-intensity, high-frequency currents	Large diameter Aβ fibers to elicit segmental analgesia	Dermatological manifestations, labor pain
Acupuncture-like TENS	High-intensity, low-frequency	Small diameter Aδ fibers to elicit extrasegmental analgesia	Long-standing deep pain, postoperative pain
Intense TENS	High-intensity, high-frequency	Small diameter Aδ fibers to elicit extrasegmental analgesia and peripheral nerve blockade	Pain associated with cancer, counterirritant for wound dressing and suture removal
Burst Mode TENS	Variable-intensity bursts of low and high-frequency currents	Large diameter Aβ and small diameter Aδ fibers to elicit bursts of segmental and extrasegmental analgesia	Vascular Resistance Pain
Modulated TENS	Variable	Variable	Soft tissue pain

Clinical application

Patient Selection

Selecting suitable patients for TENS therapy is crucial to achieving effective pain management in treating PHN. A variety of patients can be appropriate candidates for TENS, particularly those who have not responded adequately or have experienced adverse effects from conventional PHN medications, such as antivirals, TCAs, gabapentinoids, opioids, or local anesthetic patches, are suitable candidates for TENS. TENS in combination with pregabalin has been shown to reduce PHN pain more than pregabalin alone (p<.02), demonstrating its value as an adjunctive treatment [49]. For patients who have shown adverse effects from conventional medications for PHN, TENS may be a viable stand-alone treatment. One retrospective study found TENS alone to be better at reducing pain severity during acute HZ and incidence of PHN compared to antivirals (Acyclovir 800 mg 5x/day for one week or Famciclovir 1000 mg 3x/day for one week) (Chi-square test: P=.024) [50]. Additionally, the main contraindications for TENS therapy are the presence of implanted pacemakers and skin malignancies [51]. Therefore, most patients will be able to utilize this treatment safely.

Device Selection and Settings

There are many different types of TENS devices and settings, but the two most common application modes used in clinical practice are conventional TENS (high frequency; >80 Hz and pulse width < 150 μsec) and acupuncture-like TENS (low frequency; <10 Hz and pulse width > 150 μsec). There is no consensus on which frequency is more effective. Patient preference and response to different stimulation settings are highly individualized, but they should produce a strong, non-painful sensation for the patient [52,53]. Evidence shows that tolerance can develop, causing patients to adjust settings over time [54]. Modern devices allow customization of pulse width, frequency, and intensity, which can match the patient's pain profile and tolerance [34].

Integration into Clinical Practice

Optimal pain management strategies are holistic, combining pharmacologic treatments with self-management techniques such as TENS, physical activity, pain education, and lifestyle adjustments [55]. Clinically, it is recommended that TENS treatment strategies be personalized to the patient's needs. This includes adjusting the electrical characteristics of the currents according to personal comfort and efficacy. A recent meta-analysis of 381 randomized controlled trials involving 24,532 participants suggested that the quality of the TENS sensation, rather than the electrical parameters, is essential for its efficacy. This supports the guidelines for clinicians to advise patients to administer TENS on or adjacent to the painful area with settings that are comfortable and effective for them [55]. Consequently, patients will have to use a trial-and-error approach to adjust parameters to optimize pain relief [56]. This makes patient education and support integral to successful TENS therapy. Educating patients about TENS device settings, electrode placement, and realistic pain relief expectations can improve treatment satisfaction and effectiveness. Regular follow-ups can further enhance this, ensuring patients are confident administering TENS therapy. Overall, integrating TENS, or other neuromodulatory techniques [57], into the management of PHN or other neuropathic pain syndromes provides a non-invasive, patient-controlled, cost-effective method to alleviate pain, making it a unique alternative treatment.

## Conclusions

The present investigation focuses on four randomized control trials that explore the safety and efficacy of using TENS as a treatment for PHN. All these studies showed a statistically significant reduction in the intensity of neuropathic pain in patients afflicted with PHN with included or exclusive use of TENS treatment without any reported adverse effects. TENS is currently contraindicated in patients with a history of epilepsy or cardiac pacemaker placement or those who are currently pregnant, which can limit its therapeutic potential for all afflicted patients. More research should be conducted into the efficacy and safety of TENS for treating PHN to justify and cement its role as a mainstay treatment for this condition.
